# Ink‐Printed Thermoelectric Materials and Devices: Material Preparation, Fabrication Techniques, and Applications

**DOI:** 10.1002/smsc.70317

**Published:** 2026-06-13

**Authors:** Juxiang Shao, Lin Li, Ming Yang, Jiali Huang, Na Zhang, Supree Pinitsoontorn, Qiang Sun, Hongchun Luo, Hao Wu

**Affiliations:** ^1^ Key Laboratory of Computational Physics of Sichuan Province Yibin University Yibin Sichuan China; ^2^ Department of Stomatology The First Medical Centre Chinese PLA General Hospital Beijing China; ^3^ Department of Physics Faculty of Science Khon Kaen University Khon Kaen Thailand; ^4^ State Key Laboratory of Oral Diseases National Center for Stomatology National Clinical Research Center for Oral Diseases West China Hospital of Stomatology Sichuan University Chengdu Sichuan China

**Keywords:** printing, thermoelectric inks, thermoelectric materials

## Abstract

Thermoelectric (TE) materials, which enable the direct conversion between thermal and electrical energy, represent a promising solution for sustainable energy technologies. While significant improvements in the figure of merit (*ZT*) have been achieved over the past two decades, a pressing need remains for scalable synthesis and flexible manufacturing methods to fabricate high‐performance devices. Additive manufacturing via printing provides precise geometric control and scalability for fabricating high‐performance TE devices. The properties of these printed devices are largely determined by the TE inks employed. This review systematically summarizes recent progress in TE inks, focusing on three key areas: material preparation, including TE ink formulation and binding strategies; processing techniques, such as inkjet printing and screen printing; and applications, highlighting advanced printed TE devices for energy harvesting, thermal management, and sensing. Given recent progress, this review establishes a framework for future development of printed TEs. Finally, we discuss emerging research and applications, opportunities, and suggest that the fundamental principles of TE ink development could find broader applications in biomedical fields such as tissue repair and disease treatment.

## Graphical abstract

Ink‐printing technique is promising in fabricating thermoelectric materials and devices for advanced wearable electronics applications.

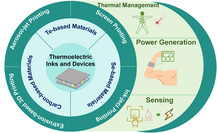

## 1 Introduction

Thermoelectric (TE) materials enable direct conversion between heat and electricity, hold significant potential for sustainable energy harvesting, advanced sensing, and solid‐state cooling, making them ideal for applications ranging from industrial systems to personal health management [1, 2, 3]. Despite significant progress in enhancing the material efficiency, quantified by the dimensionless figure of merit (*ZT*), a considerable gap remains between laboratory achievements and commercial device implementation [4]. Conventional manufacturing techniques, such as hot pressing and melting [5, 6, 7, 8, 9] they are often incompatible with complex geometries and generate significant material waste, thereby limiting the technology's broader adoption. Additive manufacturing (AM), or 3D printing, offers a promising solution to these fabrication challenges [10, 11, 12, 13]. In this approach, the layer‐by‐layer deposition approach provides exceptional design freedom, reduces waste, and enables scalable production [14, 15]. It is particularly suited for creating conformal TE generators (TEGs) that can be integrated into devices or curved surfaces, which is critical for developing wearable TE devices for bioelectronics [16, 17, 18].

Ink printing represents a versatile AM approach capable of processing diverse material systems, including pure polymers, organic/inorganic composites, and inorganic materials, to fabricate complex structures through layer‐by‐layer deposition [19, 20]. This technique encompasses various methods such as screen printing, dispenser printing, aerosol jet printing (AJP), and inkjet printing [21, 22]. It offers significant advantages, including operational simplicity, mass production capability, design flexibility, and broad material compatibility [23, 24, 25]. The layer‐wise deposition process enables precise control over geometry and thickness, facilitating enhanced flexibility and optimized TE performance in printed devices. However, the power generation performance of inkjet‐printed TE devices currently lags behind conventionally fabricated counterparts [26, 27]. This performance gap is intrinsically linked to the properties of the TE inks, which present a critical materials challenge. These inks must simultaneously exhibit optimal electrical transport characteristics in their final solid state and possess key ink properties, including colloidal stability, rheological behavior (e.g., viscosity, storage, and loss moduli), and the appropriate formulation of solvents, binders, and additives [28, 29]. Therefore, developing innovative ink formulations that integrate optimized carrier transport with these properties remains essential for achieving uniform deposition, shape fidelity, and ultimately high performance in printed TE devices [21, 30, 31, 32].

**SCHEME 1:**
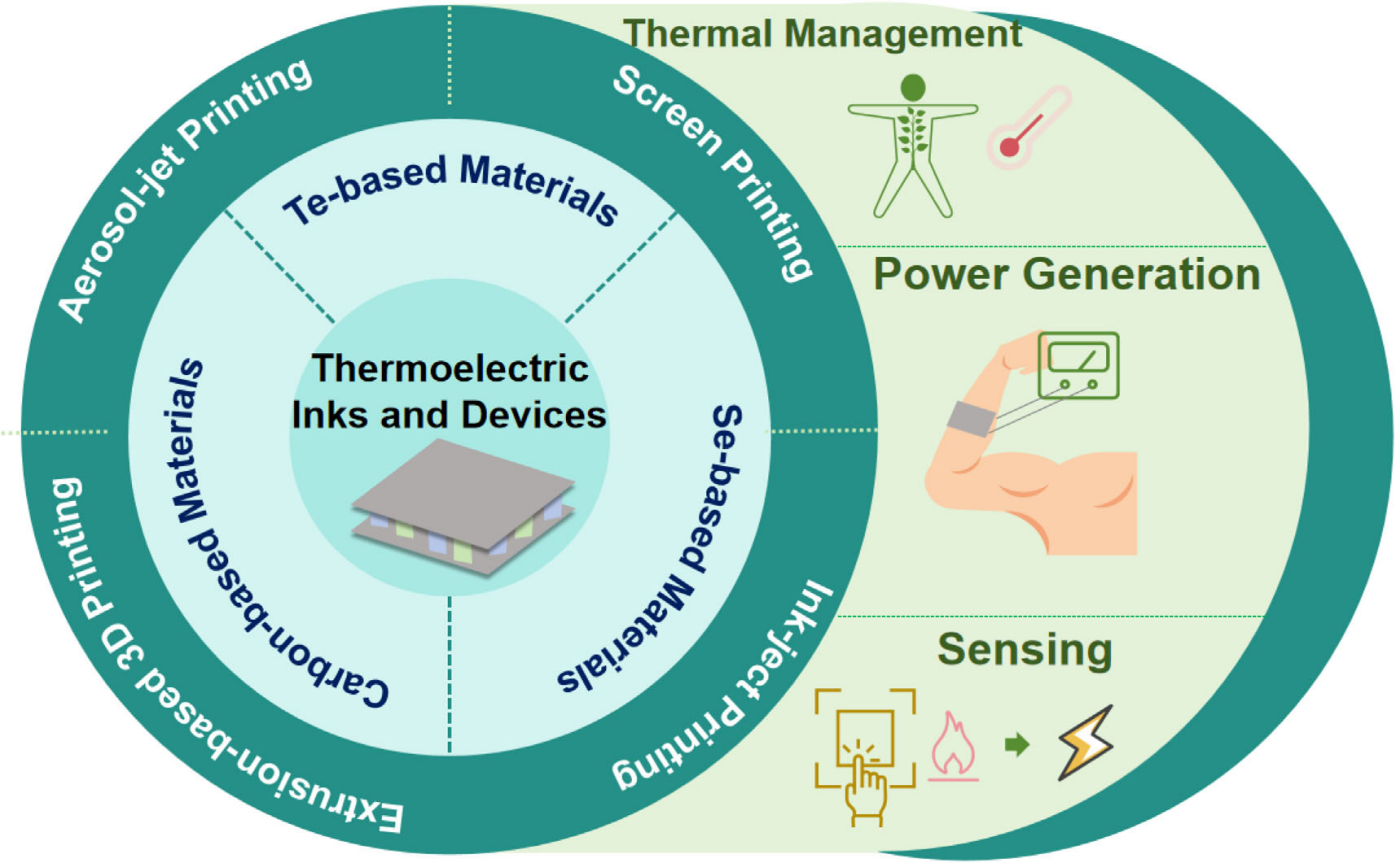
Classification of TE ink materials, binding strategies, preparation techniques, and application areas.

This review systematically investigates recent progress in TE inks, focusing on three critical domains: material preparation, involving the characterization of inorganic/organic TE substances and their binding mechanisms; fabrication techniques, exploring printing methods for creating TE materials and devices in different scales and dimensions, showcasing advanced devices for energy harvesting, thermal management, and sensing [33]. By establishing fundamental links between ink composition, processing parameters, and device performance, this work provides a structured framework for developing printed TEs. Finally, we identify key challenges and future research directions, suggesting that these ink development principles may also benefit biomedical applications, including wound repair and regeneration (Scheme 1).

## 2 Basic Principles of TEs

TE materials can realize direct conversion between thermal and electrical energy, underpinning applications in power generation, cooling, and sensing. Despite extensive research, achieving high conversion efficiency remains a significant challenge. This efficiency is quantified by the *ZT*, which is governed by the Seebeck coefficient (*S*), electrical conductivity (*σ*), thermal conductivity (*κ*), and absolute temperature (*T*), as defined in Equation (1) [1, 2, 9, 34].

(1) $$ZT=S^{2}\sigma T/\kappa$$

where *κ* = *κ*_e_ + *κ*_L_, *κ*_e_ is the electron heat conduction coefficient, and *κ*_L_ is the lattice heat conduction coefficient, *T* is the average device operating temperature, and *S* ^2^ *σ* is defined as the power factor (*PF*). Optimizing these properties is essential for maximizing power output (*P*), and their combined performance is a key metric for evaluating TEGs.

The design of TE devices is guided by principles that are highly compatible with the adaptability of ink printing techniques. In particular, for TEGs, the primary goal is to maximize the *P*, which is dictated by the material's conversion efficiency and the heat flux through the device, according to the relationship *P* = *ηQ*. The efficiency *η* depends on the temperature differences across the TE unit, which can be evaluated by using Equation (2) [28, 35].

(2) $$\text{η}={\text{η}}_{r}\frac{{\Delta T}_{\mathrm{TE}}}{T_{h}}$$

Then, *P* can be expressed as Equation (3)

(3) $$\text{P}={\text{η}}_{r}\frac{{\Delta T}_{\mathrm{TE}}}{T_{h}}Q$$

where ${\text{η}}_{r}$ is the reduced device efficiency, a function of the *ZT*. As a consequence, ${\text{η}}_{r}$ can be expressed as Equation (4) [35],

(4) $$\eta_{r}=\frac{\sqrt{1+ZT}-1}{\sqrt{1+ZT}+\frac{T_{c}}{T_{h}}}$$

Therefore, a higher *ZT* value directly enhances conversion efficiency and *P*, necessitating the development of high‐performance TE materials. For printed devices, this objective depends critically on the preparation of high‐quality TE inks. These inks must be formulated to exhibit not only the fundamental material properties for high TE performance, specifically, a high *S*, high *σ*, and low *κ* [1, 19, 36], but also the appropriate rheological characteristics for the printing process itself. In the following section, the current process for preparing inks using TE materials and their binding strategy will be discussed.

## 3 TE Inks

This section reviews the materials and methods of creating TE inks. Various materials with promising TE properties are used in ink formulations. Reported examples include inorganic compounds (e.g., Bi_2_Te_3_ [37], Sb_2_Te_3_ [38], Cu_2_Se [39], Ag_2_Te [37], carbon‐based materials like carbon nanotubes (CNTs) [40]. Table 1 presents representative high‐performance material systems for TE inks, together with their printing methods and post‐processing treatments. These systems were either for their excellent performance near room temperature or their intrinsic flexibility, both of which are essential for applications such as wearable electronics [52].

**TABLE 1:** Representative high‐performance material systems for TE inks.

Main material composition	Semiconductor type	*ZT* (key TE performance)	Binder	Printing method	Post‐processing
PbTe (Pb_0.98_Na_0.02_Te) [41]	P	≈1.0	Te	Screen printing	Cold pressed then annealed in a tube furnace
Sb_2_Te_3_ [42]	P	—	—	3D printing	Cold pressed then thermal sintered
Bi_2_Te_3_ (Bi0.4Sb1.6Te3) [43]	P	≈1.0	Te	Screen printing	Sintered using a tube furnace under an inert environment
Ag_2.1_Te [37]	N	≈0.19	—	Inkjet printing	Dried in a vacuum oven and then hot pressed
Bi_2_Se_3_ [44]	N	≈0.1	PPGDGE/D.E.R.736	Screen printing	Hot‐pressing
Cu_2_Se [45]	N	≈0.15	PVP	Screen printing	Sintered in a vacuum oven
Ag_2_Se [46]	N	≈1.3	—	3D printing	Dried in an oven, then annealed
SnSe [47]	P	≈1.7	Carboxymethylcellulose	pseudo‐3D printing	Sintered by SPS
InSe [48]	N	—	Organic binder(CAP + DAA)	Screen printing	Dried in a hot‐air oven
CNT [49]	N	—	—	Screen printing	—
PEDOT:PSS [50]	P	—	Sb_2_Te_3_	AJP	Dried in the air
PANI/Graphite [51]	P	—	Cellulose acetate	Screen printing	—

Conventional methods for fabricating TE materials, such as hot pressing [53] and spark plasma sintering [54], are well‐established. However, unlike direct patterning methods (e.g., printing), these consolidation approaches do not directly enable the fabrication of complex device geometries, though they can serve as a post‐processing step for printed structures. They are primarily suited for producing dense bulk materials, and creating intricate shapes typically requires additional treatment steps, which generate substantial material waste and lead to higher costs. In contrast, printing technologies offer a pathway to more versatile and cost‐effective manufacturing. Therefore, the development of high‐quality TE inks is essential for advancing printed electronics. The following discussion will first examine the key nanomaterials used in these inks and their properties. It will then explore the binding strategies that are critical for forming dense, functional films after printing and heat treatment. Based on a review of recent literature, printable TE inks can be categorized by their primary chemical composition into selenium compounds, tellurium compounds, and carbon‐based materials.

### 3.1 TE Materials Used for Ink Preparation

#### 3.1.1 Tellurium‐Based TE Materials

**FIGURE 1:**
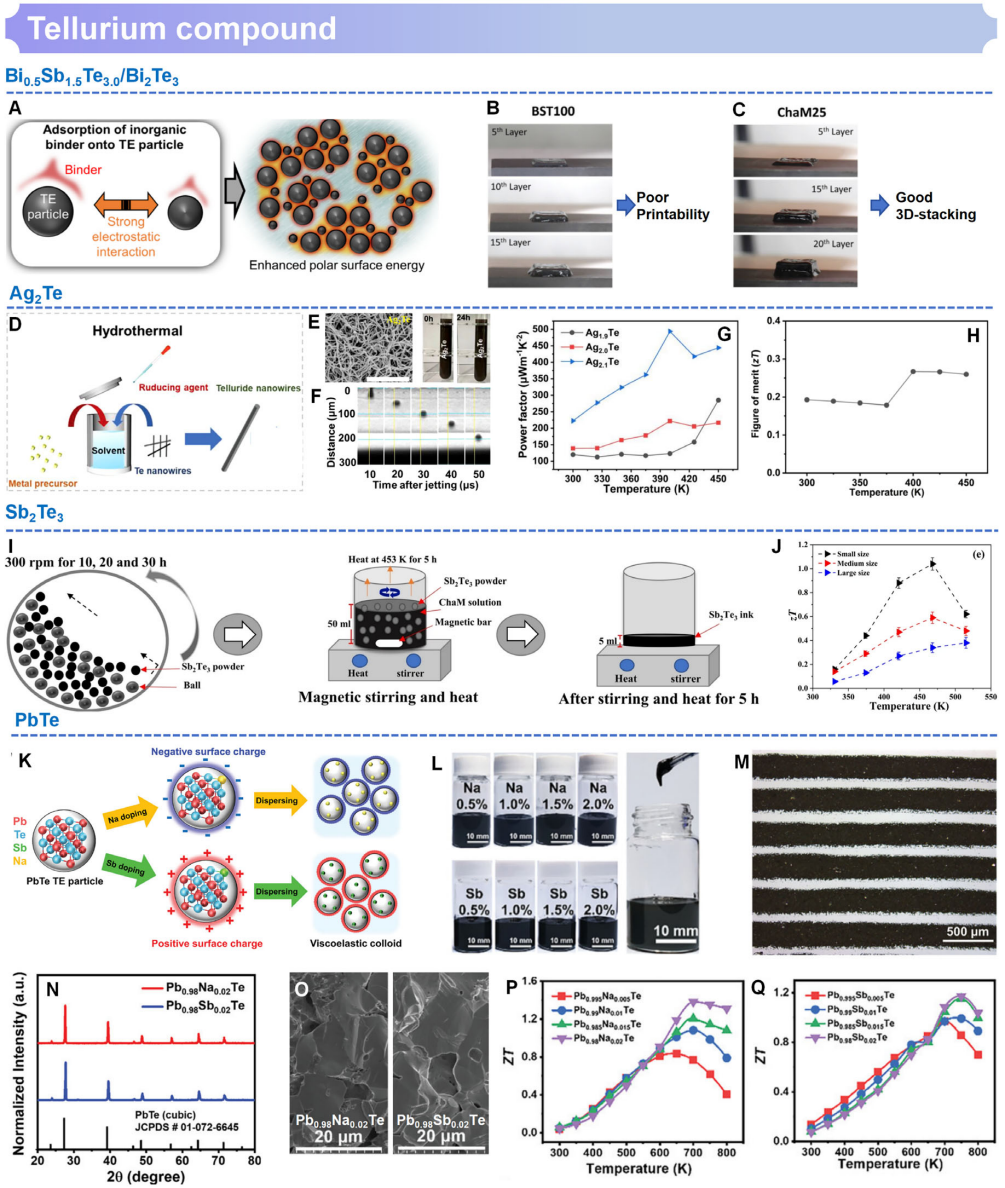
Synthesis and characterization of tellurium‐based TE inks. (A) Schematic illustration of the enhanced electrostatic interaction and polar energy of TE particles. (B,C) Design of the 3D printing process for producing a series of diagonal grid layers to assess the layer‐by‐layer stacking properties of TE inks (B), and observation of the 3D stacking behavior of BST and ChaM‐based TE inks with increasing number of printed layers (C). Reproduced with permission [58]. Copyright 2019, AIP Publishing. (D) Schematic diagram illustrating the fabrication of metal chalcogenide TE films via a templated chemical transformation. (E) SEM and TEM images of the synthesized Ag_2_Te NWs, alongside digital photographs of the stable, additive‐free inks. (F) Sequential images of the inkjet droplet formation process over a 50 µs interval. (G) Temperature‐dependent *PF* of inkjet‐printed Ag_2_Te films with different Ag/Te ratios. (H) Temperature‐dependent *Z* *T* of the optimized Ag_2.1_Te film, assuming a constant lattice thermal conductivity (*κ*_l_) from 300 to 450 K. Reproduced with permission [37]. Copyright 2023, John Wiley & Sons. (I) Schematic of the preparation process for Sb_2_Te_3_ screen‐printing inks. (J) Temperature‐dependent *ZT* for films prepared with small, medium, and large particle sizes. Reproduced with permission [38]. Copyright 2024, Springer Nature. (K) Schematic illustrating electronic doping‐induced surface charges on PbTe particles. (L) Photograph of Pb_1‐*x*_M*_x_* Te TE inks with varying doping contents (M = Na or Sb, 0.5% ≤ *x* ≤ 2%). (M) Optical microscopy image of printed lines of Pb_0.98_Na_0.02_Te ink. (N) XRD patterns of 3D‐printed Pb_0.98_Na_0.02_Te and Pb_0.98_Sb_0.02_Te samples; vertical lines mark peaks corresponding to bulk cubic PbTe (JCPDS: 01‐072‐6645). (O) SEM images of the 3D‐printed samples reveal large grains tens of micrometers in size. (P,Q) Temperature‐dependent TE *ZT* of Na‐doped (*p*‐type) and Sb‐doped (*n*‐type) PbTe. Reprinted with permission from [41]. Copyright 2018, American Chemical Society.

Since its discovery in the 1950s, Bi_2_Te_3_ has been the subject of extensive research, leading to numerous synthetic methods. Bi_2_Te_3_‐based materials are among the most efficient TE systems at room temperature and are widely used in commercial applications [55]. Their properties can be finely tuned through doping and alloying. For instance, *n*‐type materials can be created by doping with selenium (e.g., Bi_2_Te_2.7_Se_0.3_), while alloying with Sb_2_Te_3_ produces the high‐performance *p*‐type material Bi_0.5_Sb_1.5_Te_3_ [56]. These compounds exhibit peak performance in the temperature range of 300–500 K, making them ideal for applications such as low‐grade waste heat recovery and solid‐state cooling devices. Sung Hoon Park et al. demonstrated 3D printing of shape‐conformable TE materials using viscoelastic, all‐inorganic Bi_2_Te_3_‐based inks [57]. Their synthesis employed Sb_2_Te_3_ chalcogenidometallate (ChaM) ions as inorganic binders for Bi_2_Te_3_ particles. Then Jae Sung Son, Han Gi Chae et al. reported inks using BiSbTe‐based TE particles [58]. In this work, the rheological behavior of all‐inorganic inks was analyzed to assess printability and 3D structural retention with respect to the ChaM content. Figure 1A shows the schematic presentation of enhanced electrostatic interaction and polar energy of TE particles. As compared in Figure 1B,C, the introduction of ChaM can significantly improve the formation ability of inks in printing. Then the subsequent studies by Fredrick Kim et al. further extended this strategy toward advanced printing techniques, including three‐dimensional fabrication and scalable device integration [59]. The phase purity of the printed materials was confirmed by X‐ray diffraction (XRD), with patterns for the *n*‐type and *p*‐type samples matching the standard profiles for Bi_2_Te_3_ and Bi_0_._5_Sb_1_._5_Te_3_, respectively. These materials also showed excellent TE performance, achieving dimensionless *ZT* values of 0.9 for *p*‐type and 0.6 for *n*‐type at room temperature, which are comparable to bulk material values.

Ag_2_Te is a high‐performance *n*‐type TE material, exhibiting notably TE performance near room temperature [60]. Its crystal structure is phase‐dependent, transitioning from a monoclinic (*β*‐Ag_2_Te) structure at lower temperatures to a cubic (*α*‐Ag_2_Te) phase above approximately 145°C [61]. A key characteristic of Ag_2_Te is its intrinsically ultralow *κ*, which is crucial for TE efficiency as it reduces heat conduction while preserving electrical transport. This combination of properties yields an excellent *ZT* [62]. Furthermore, its earth‐abundant composition and compatibility with scalable processing methods, such as inkjet printing, make it highly promising for developing flexible TEGs.

In a relevant study, Du et al. presented an economical and scalable inkjet printing method for fabricating high‐performance flexible TE devices [37]. This approach utilized metal chalcogenide nanowires (NWs) dispersed in ethanol to create additive‐free inks. Using Ag_2_Te as a representative material, the team produced fully printed flexible films that achieved a high *PF* of 493.8 µW m^−1^ K^−2^. Devices made from these films reached a maximum power density of 0.9 µW cm^−2^ K^−2^, a performance that surpasses many other inkjet‐printed TEs. The authors note that this versatile printing platform can also be applied to other materials, such as Cu_7_Te_4_ and Bi_2_Te_2_._7_Se_0_._3_, facilitating scalable manufacturing for next‐generation wearable applications. The experimental process and results are detailed in Figure 1. Figure 1D schematically illustrates the fabrication of the metal chalcogenide films via a templated chemical transformation. The successful synthesis of one‐dimensional Ag_2_Te NWs and the stability of the resulting inks are confirmed by scanning electron microscopy (SEM) and transmission electron microscopy (TEM) images and digital photographs in Figure 1E. Figure 1F captures the reliable jetting process, indicating good ink quality. Finally, the TE performance is quantified in Figures 1G,H, which present the *PF* for different Ag/Te ratios and the high *ZT* value for the optimized Ag_2_._1_Te film, demonstrating the exceptional performance of the printed material.

Sb_2_Te_3_ is a classic *p*‐type TE material, recognized for its high performance near room temperature [63]. It possesses a rhombohedral crystal structure (space group R$\bar{3m}$), characteristic of the tetradymite family. This structure is composed of quintuple layers (Te–Sb–Te–Sb–Te) held together by weak van der Waals forces along the *c*‐axis, while strong covalent bonds dominate within the layers [64]. This anisotropic, layered architecture results in intrinsically low *κ*_l_ due to effective phonon scattering at the layer boundaries.

In a representative study, Surasak Ruamruk et al. fabricated a high‐performance Sb_2_Te_3_ thick film using a screen‐printing technique [38]. The TE ink was prepared by mixing Sb_2_Te_3_ powders of different sizes with a ChaM‐based solution. Figure 1I shows a schematic of this ink preparation process. The resulting material's morphology was examined using SEM. The TE performance of these films is presented in Figure 1J. The data clearly demonstrate that the film fabricated with the smallest powder size achieved a remarkable maximum *ZT* value of 1.04 at 468 K. This performance is more than three times greater than that of films using larger particles, underscoring the critical role of microstructural control and particle size in optimizing TE properties.

PbTe‐based compounds rank as top‐performing TE materials within the 500–800 K window. The high‐symmetry cubic structure, strong anharmonicity, and complex band architecture of PbTe simultaneously enable a high *S*, excellent *σ*, and suppressed *κ* [41]. Jae Sung Son et al. reported the development of extrusion‐based 3D printing of *n*‐type and *p*‐type PbTe materials and proposed a design for a power‐generating TE tube with customized tubular PbTe legs, representing a frontier work in the field of printed PbTe‐based TEs. As shown in Figure 1K, doping‐induced surface charges on PbTe particles significantly improve the viscoelasticity of the inks without the need for additives, thereby leading to precise shape and dimension engineering of 3D bulk PbTe. Figure 1L presents Pb_1‐*x*_M_*x*_Te TE inks with various doping contents (M = Na or Sb, 0.5% ≤ *x* ≤ 2%). Figure 1M shows an optical microscopy image of printed lines of Pb_0.98_Sb_0.02_Te ink, and the XRD patterns of 3D‐printed Pb_0.98_Na_0.02_Te and Pb_0.98_Sb_0.02_Te samples are displayed in Figure 1N. The vertical lines indicate the peaks corresponding to bulk cubic PbTe. Figure 1O shows SEM images of the 3D‐printed Pb_0.98_Na_0.02_Te and Pb_0.98_Sb_0.02_Te samples, revealing large grains with sizes of several tens of micrometers. Figure 1P,Q display the temperature‐dependent TE *ZT* of Na‐doped and Sb‐doped PbTe. Owing to the rheological design of the inks, the 3D printing process achieved the desired functionality for dimension and shape engineering of PbTe TE legs. Consequently, the 3D‐printed materials exhibited particularly high *ZT* values of 1.4 for the *p*‐type and 1.2 for the *n*‐type [65].

#### 3.1.2 Selenium‐Based TE Materials

**FIGURE 2:**
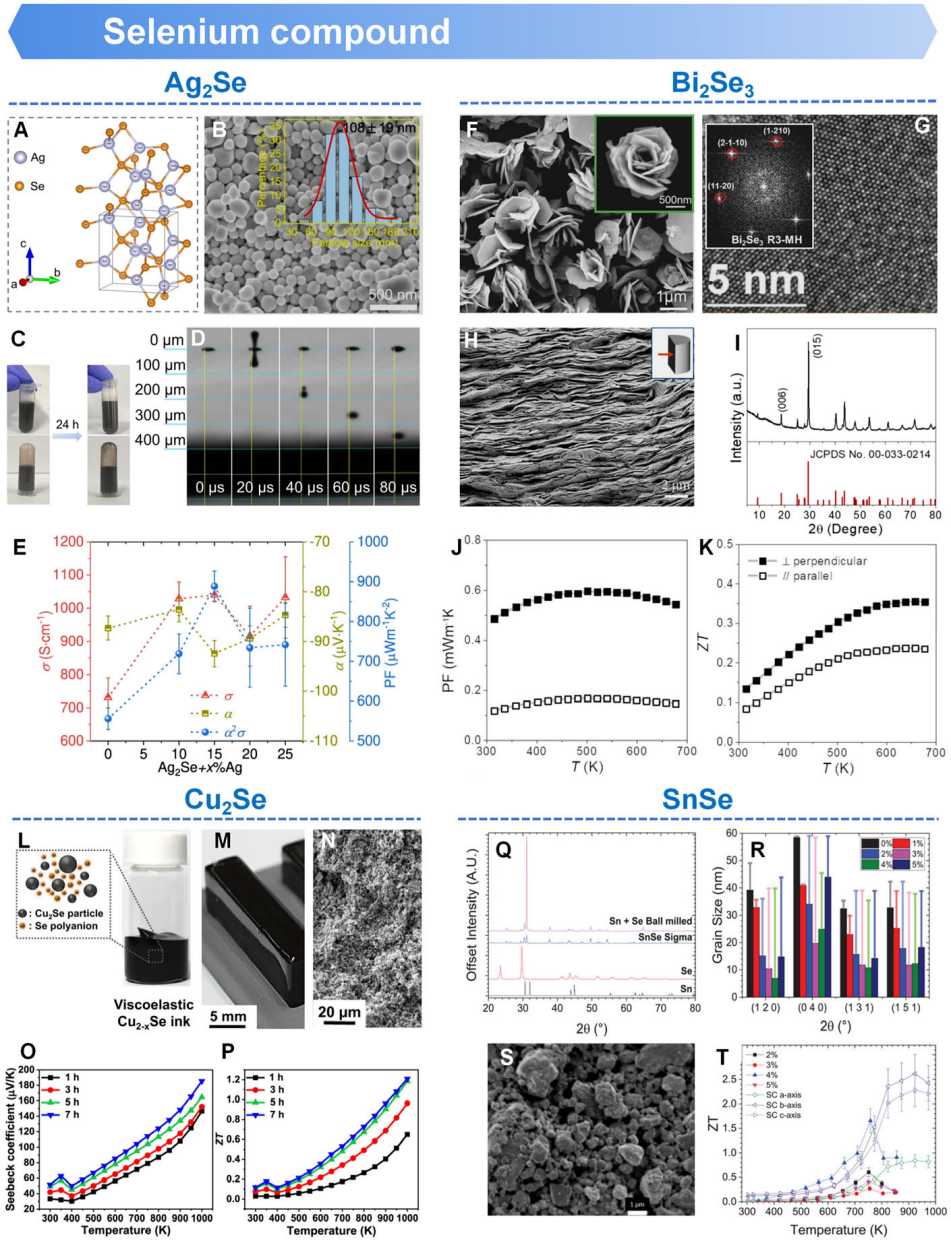
Synthesis and characterization of selenium‐based TE inks. (A) Schematic atomic structure of Ag_2_Se. (B) SEM image of Ag_2_Se nanoparticles, with an inset showing the particle size distribution. (C) Digital photographs of the Ag_2_Se ink in normal and inverted states before and after 24 h, demonstrating its stability. (D) Sequential images showing the jetting cycle of a 10 pL Ag_2_Se ink droplet over a fixed interval. (E) Room‐temperature electrical conductivity (*σ*), *S*, and *PF* as a function of Ag content in the ink. Reproduced with permission [68]. Copyright 2024, Springer Nature. (F) SEM and (G) high‐resolution TEM (HRTEM) micrographs of Bi_2_Se_3_ nanoflakes. (H) Cross‐sectional SEM image of a consolidated Bi_2_Se_3_ pellet. (I) XRD pattern of the Bi_2_Se_3_ pellet. (J) *PF* and (K) *ZT* of Bi_2_Se_3_ nanomaterials measured along two directions relative to the pressing axis. Reproduced under terms of the CC‐BY 4.0. license [44]. Copyright 2023, Yuan Z. et al*.*, published by MDPI. (L) Digital photograph of an all‐inorganic Cu_2‐*x*_Se ink. (M) Digital photograph of 3D‐printed Cu_2‐*x*_Se structures. (N) SEM image of the fractured surface of a 3D‐printed Cu_2‐*x*_Se sample before sintering. (O) *S* and (P) *ZT* values for Cu_2‐*x*_Se samples sintered at 873 K for different durations. Reproduced with permission [39]. Copyright 2021, Springer Nature. (Q) Comparison of ball‐milled SnSe powder with commercial SnSe powder and the starting materials, prepared for SnSe inks. (R) Grain size distributions derived from four diffraction peaks: columns represent uncured samples, and bars represent cured samples, across all binder concentrations. (S) SEM image of SnSe containing 4% binder after curing. (T) Comparison of *ZT* with that of single‐crystal SnSe. Reproduced with permission [47]. Copyright 2019, John Wiley & Sons.

Ag_2_Se is a narrow‐bandgap semiconductor recognized as a high‐performance TE material, especially near room temperature. It achieves a high *ZT* due to its intrinsically low *κ*_l_ and favorable electrical transport properties [66, 67]. Wan Jiang et al. demonstrated a scalable inkjet printing method for fabricating flexible Ag_2_Se TE devices [68]. Through optimization of ink formulation and printing parameters, they achieved large‐area, microscale patterning. Figure 2A,B presents the atomic structure and SEM image of the Ag_2_Se nanoparticles, respectively, with the inset confirming a uniform particle size distribution. The ink's stability is evidenced by its unchanged state after 24 h (Figure 2C), and its printability is confirmed by a stable droplet jetting cycle (Figure 2D). Consequently, the printed (00l)‐textured Ag_2_Se films yielded an exceptional *PF* of ≈1100 μW m^−1^ K^−2^ at 377 K, achieved through microstructural engineering (Figure 2E). This approach enables the cost‐effective manufacturing of flexible power sources for wearable electronics, such as body heat harvesting devices. In addition, Qinfei Ke et al. reported the fabrication of flexible TE films, including Ag_2_Se/methylcellulose, PEDOT:PSS@Ag_2_Se/methylcellulose, and PVP@Ag_2_Se/methylcellulose, using a scalable and cost‐effective direct‐ink printing method. The authors attribute the enhanced performance to the microstructural features formed during the fabrication process. Heterointerfaces, pores, grain boundaries, and dislocations within the composite films contribute synergistically to a higher *S* and increased *σ*, while simultaneously reducing the lattice *κ* [19].

Sb_2_Se_3_ is an emerging *p*‐type TE material with considerable potential for mid‐temperature applications. It features an orthorhombic crystal structure consisting of one‐dimensional [Sb_4_Se_6_]_*n*_ ribbons. These ribbons create a highly anisotropic structure with strong covalent bonding within the ribbons and weak van der Waals forces between them, resulting in an intrinsically low *κ*_l_. In a related study, Zicheng Yuan et al. fabricated a five‐pair TE module by screen‐printing commercial Sb_2_Te_3_ (*p*‐type) and synthesized Bi_2_Se_3_ nanoflakes (*n*‐type) [44]. Figures 2F,G show SEM and HRTEM images confirming the nanoflake morphology and high crystallinity of the Bi_2_Se_3_. Figure 2H presents a cross‐sectional SEM image of the sintered pellet, and Figure 2I shows the XRD pattern confirming crystal structure and orientation. The TE performance, displayed in Figure 2J,K, reveals anisotropic *PF* and *ZT* values relative to the pressing direction. The device achieved an open‐circuit voltage of 0.11 V and a maximum output power of 9.5 μW at a temperature difference of 109.5 K, significantly outperforming a module made with commercial Bi_2_Se_3_ powder and highlighting the advantage of nanoengineered materials for flexible electronics.

Cu_2_Se is a promising TE material composed of earth‐abundant, low‐toxicity elements [69], attributed to the liquid‐like motion of copper ions within a rigid selenium sublattice, which effectively scatters heat‐carrying phonons [70]. This property, combined with decent *σ*, contributes to a high *ZT*. However, challenges with elemental migration and stability under operational conditions remain active areas of research for this material. Seungjun Choo et al. report the design of cellular TE architectures via extrusion‐based 3D printing of Cu_2_Se [39]. A key advance was the development of an organic binder‐free ink, with desirable viscoelasticity achieved using an inorganic Se_8_^2−^ polyanion additive. Figure 2L,M shows the prepared ink and a 3D‐printed structure, respectively. The microstructure of a printed sample before sintering is shown in Figure 2N. The TE properties of samples sintered for different durations are presented in Figure 2O,P. The sample sintered for 7 h achieved a maximum *ZT* of 1.21 at 1000 K, a value comparable to previously reported bulk Cu_2_Se [71].

Bi_2_Te_2_._7_Se_0_._3_, alloying of Bi_2_Te_3_ with selenium to form, is a well‐established method for optimizing *n*‐type TE materials. This compound benefits from nanostructuring, which further reduces its lattice *κ*. Priyanshu Banerjee et al. enhanced the performance of *n*‐type chitosan‐Bi_2_Te_2.7_Se_0.3_ composite films by employing heterogeneous grains and applying mechanical pressure [72]. In this work, the optimized composite achieved a *σ* of 200 ± 7 S cm^−1^, a *S* of −201 ± 6 µV K^−1^, and a *PF* of 808 ± 69.7 μW m^−1^ K^−2^. With a *κ* of 0.6 W m^−1^ K^−1^, a room‐temperature *ZT* of 0.4 was achieved, demonstrating a competitive and sustainable fabrication route for printable TEs.

SnSe is a promising TE material, particularly in the medium‐temperature range. At 300 K, bulk SnSe exhibits an orthorhombic crystal structure belonging to the Pnma phase (*a* = 11.49 Å, *b* = 4.44 Å, *c* = 4.14 Å), whereas at high temperatures (750–800 K) it transforms to the Cmcm structural phase. In recent years, SnSe has been explored as a matrix material for printing applications. In 2019, Matthew R. Burton, Matthew J. Carnie, and their colleagues developed a pseudo‐3D printing technique to fabricate bulk SnSe TE elements [47], enabling the production of TE generators in a standard configuration (Figure 2Q,T). Among the fabricated samples, an element printed from an ink containing 4% organic binder achieved the highest performance (Figure 2S), with a *ZT* value of 1.7 (±0.25) at 758 K, as displayed in Figure 2T. Subsequently, the same group fabricated bulk n‐type SnSe elements by using Bi as a dopant. Various Bi doping levels were investigated and characterized over a wide temperature range and across multiple thermal cycles [73]. Additionally, they explored the use of an inorganic binder (sodium metasilicate) combined with reusable molds for rapid printing [74]. In addition, InSe has also been explored for printed TEs. Manasa R. Shankar et al. synthesized Bi/Te‐codoped InSe powders via a conventional solid‐state reaction method and subsequently fabricated FTEGs using a facile, scalable screen‐printing technique [48].

#### 3.1.3 Carbon‐Based and Organic TE Materials

**FIGURE 3:**
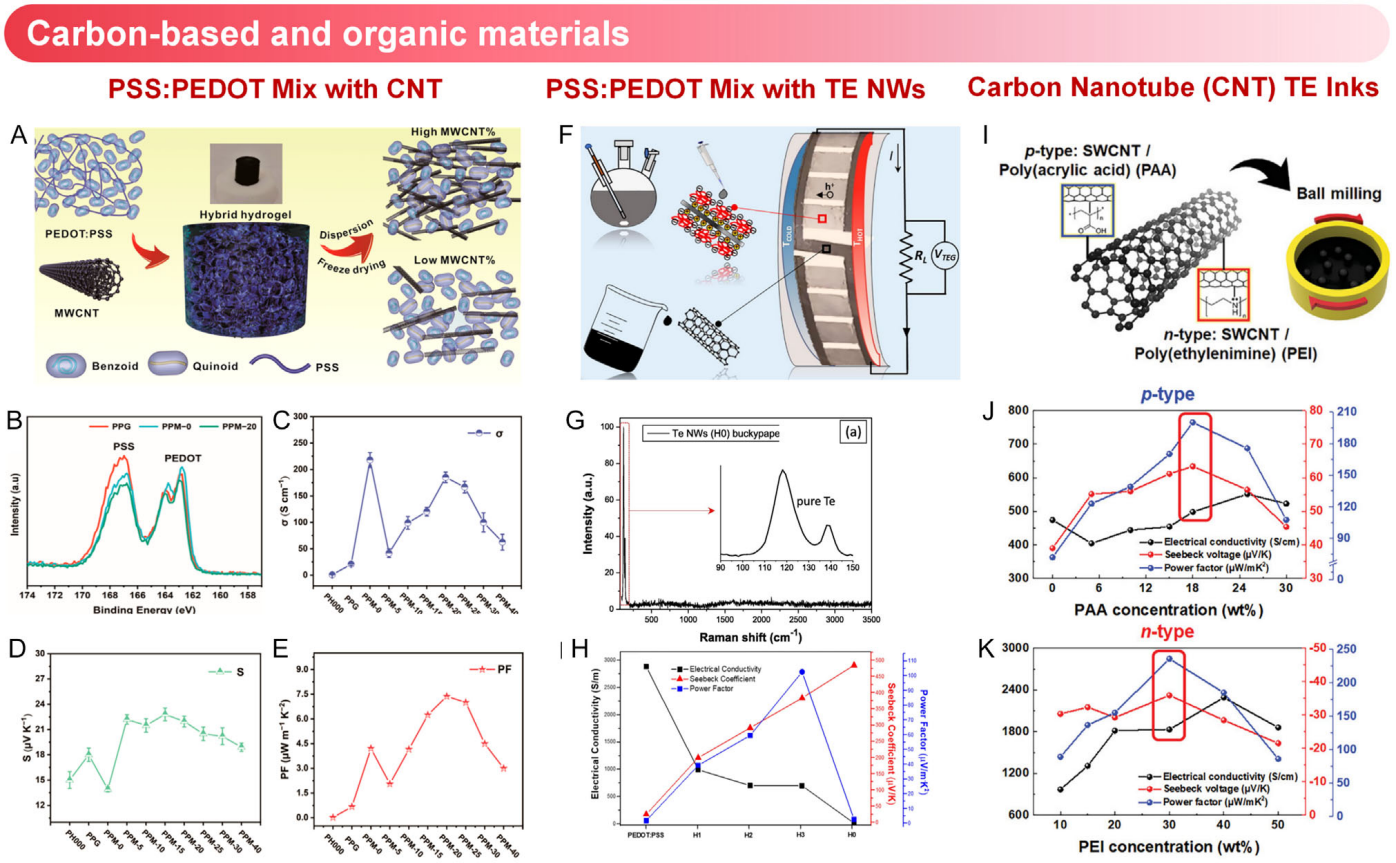
Synthesis and characterization of carbon‐based and organic TE inks. (A) Schematic illustration showing the evolving distribution of PEDOT:PSS and MWCNTs during composite formation and representative (B) XPS spectra. (C–E) Systematic evaluation of room‐temperature TE properties: (C) *σ*, (D) *S*, and (E) calculated *PF*. Reproduced with permission [40]. Copyright 2025, American Chemical Society. (F) Schematic diagram illustrating the synthesis steps for TeNW@PEDOT:PSS hybrid inks and films. (G) Spectrum characterization of the obtained inks. (H) In‐plane TE properties: *σ*, *S*, and *PF*. Reproduced with permission [14]. Copyright 2024, the Royal Society of Chemistry. (I) Schematic of the preparation process for pre‐doped *p*‐type and *n*‐type CNT inks. TE properties of CNT inks as a function of dopant concentration: (J) PAA‐doped *p*‐type and (K) PEI‐doped *n*‐type inks, showing *σ*, *S*, and *PF*. Reproduced under terms of the CC‐BY 4.0. license [26]. Copyright 2023, Li H. et al*.*, published by MDPI.

Poly(3,4‐ethylenedioxythiophene) polystyrene sulfonate (PEDOT:PSS) serves as a foundational polymer for flexible TE applications [75]. As a *p*‐type organic semiconductor, it offers intrinsic advantages including mechanical flexibility, low *κ*, and solution processability [76]. While its TE performance was initially modest, significant enhancements in *σ* and the *S* have been achieved through secondary doping and post‐treatment with solvents or acids. These optimizations yield improved *PF* *s*, making PEDOT:PSS a leading candidate for generating electricity from low‐grade waste heat or body temperature in wearable electronics, seamlessly integrating energy conversion into flexible substrates. Recently, PSS:PEDOT polymers were explored as a major material for preparing TE inks [17]. For instance, J.K. Xu et al. developed a stretchable PEDOT:PSS‐based TE composite and formulated an ink with excellent viscoelasticity optimized for an all‐3D‐printing fabrication method, enabling high‐resolution, multimaterial patterning. The resulting all‐3D‐printed stretchable TE devices achieved stable performance under >50% strain and over 2000 stretching cycles, producing an open‐circuit voltage of ≈ 3.18 mV and power density of ≈6.78 nW cm^−2^ at a 40 K temperature gradient, sufficient to continuously power an LED by harvesting body heat during dynamic body motions. Moreover, PEDOT:PSS can be readily combined with inorganic nanostructures, e.g., CNT [40], Bi_2_Te_3_ [77], and Te nanomaterials, to form hybrid inks that synergistically enhance the TE *ZT* while maintaining mechanical flexibility. For example, composite hydrogels of PEDOT:PSS and multi‐walled (MWCNTs) can be prepared via a self‐assembling gelation process, which utilizes the direct interaction between the two components without requiring surfactants. Subsequent immersion in dimethyl sulfoxide (DMSO) and freeze‐drying yields a material that can be readily redispersed in water to form aqueous inks for direct ink writing (DIW) 3D printing, as illustrated in Figure 3A [40]. The successful formation of the composite inks is confirmed by X‐ray photoelectron spectroscopy (XPS), as shown in Figure 3B. The peak between 170–166 eV corresponds to sulfur in the PSS chains, while the doublet at 162–166 eV originates from the C—S bond in the PEDOT chains. The relative areas of these peaks are used to estimate the PEDOT‐to‐PSS ratio. Furthermore, the room‐temperature TE properties of the samples were systematically evaluated (Figure 3C–E). As the MWCNT concentration increases, the *σ* rises, but the *S* decreases slightly. This phenomenon may be attributed to intensified charge carrier scattering, potentially from enhanced phonon‐polariton interactions introduced by the MWCNTs.

The combination of organic and inorganic materials has been found to be a potential solution for achieving ambient TE energy harvesting and has been developing rapidly. George Karalis et al. demonstrate a water‐based scalable preparation method of Te NWs and hybrid TE inks utilizing PEDOT:PSS, for the fabrication of printed TEGs [14]. Figure 3F shows the Illustration of the steps followed to synthesize Te NW@PEDOT:PSS hybrid inks and films. To verify the successful preparation, Figure 3G shows the Raman spectra of synthesized Te NWs in the form of bucky‐paper film. Figure 2H shows the In‐plane TE properties: *σ*, *S*, and *PF*. The observed trend can result from two factors: PSS serves as a counterion for cationic, conductive PEDOT in the complex, and it facilitates a stable, easily processable dispersion of PEDOT:PSS polymer gel particles. Adding conductive polymers to Te NWs reduces contact resistance by forming conductive bridges across the nanograined Te NW networks.

In addition, low‐cost polyaniline (PANI) has also been explored for printing flexible TEGs due to its potential for low‐temperature applications. K.Mohan Rao et al. formulated composite inks by incorporating PANI and graphite with cellulose acetate as a resin and diacetone alcohol as a solvent [51]. A systematic investigation revealed that diacetone alcohol enhanced PANI's *σ* and increased *n*. The resulting TEG achieved a maximum *S* of 244.34 μV/K and a *P* of 4.31 nW at a temperature difference of 77 K.

CNTs are promising TE materials due to their high *σ*, excellent flexibility, light weight, and tunable electrical properties [78]. A key advantage is their processability into highly concentrated, viscoelastic inks with tunable rheology, making them suitable for fabricating three‐dimensional TEGs via DIW [79]. Figure 3I illustrates the preparation of pre‐doped *p*‐type and *n*‐type CNT inks [17]. To enable conformal 3D printing, viscoelastic inks were formulated by dispersing SWCNTs in diethylene glycol (DEG) with poly(acrylic acid) (PAA) as a *p*‐type dopant and polyethylenimine (PEI) as an *n*‐type dopant, using planetary ball milling. The resulting TE properties are shown in Figures 3J,K. For the *n*‐type ink, a dopant concentration of 30 wt% PEI yielded the highest *PF* of 235.05 µW m^−1^ K^−2^. While the absolute *S* initially increased with PEI addition, confirming *n*‐type conversion, it decreased beyond 30 wt%, consistent with the inverse relationship between the *S* and carrier concentration (*n*) at high doping levels.

### 3.2 Binding Strategies of TE Nanomaterials in Inks

In TE ink preparation, binders become necessary when the printing technique demands specific rheological properties, such as high viscosity for screen printing or sufficient storage modulus and yield stress for extrusion‐based 3D printing, that neat particle dispersions cannot provide. Binders also enhance interparticle connectivity and structural integrity prior to sintering, particularly for thick or complex architectures [80]. However, a key challenge remains: conventional binders like ethyl cellulose [81] and polyvinyl alcohol [82] offer mechanical robustness but are electrically insulating. Consequently, ink formulation must carefully balance binder content to ensure printability and film durability while minimizing adverse effects on *σ* and overall TE performance. Based on reported strategies, TE inks can be classified into three categories: those using inorganic binders, organic binders, and binder‐free systems.

**FIGURE 4:**
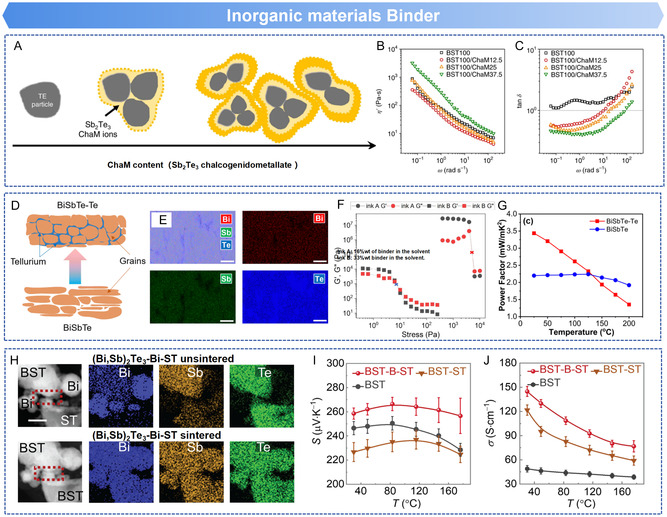
Preparing TE inks using Inorganic material binders. (A) Schematic illustrating the binding of Bi_2_Te_3_ nanoparticles by Sb_2_Te_3_ chalcogenidometallate (ChaM) ions. (B,C) Dynamic viscosity (*η*′, panel B) and loss tangent (tan *δ*, panel C) of BST100/ChaM inks with varying ChaM content. The dashed line at tan *δ* = 1 demarcates liquid‐like (tan *δ* > 1) and solid‐like (tan *δ* < 1) behavior. Reproduced with permission [59]. 2018, Springer Nature. (D) Mechanism of Te‐assisted liquid‐phase sintering in BiSbTe. (E) EDS elemental mapping of the BiSbTe‐Te composite (scale bar: 5 μm). (F) Frequency‐dependent viscoelastic properties of the ink, showing a reduced yield strength for improved printability. (G) Temperature‐dependent *PF* of the printed materials at different Te concentrations. Reproduced with permission [83]. Copyright 2024, Royal Society of Chemistry. (H) In situ TEM images and corresponding EDX elemental maps of the BST‐B‐ST ink before (top) and after (bottom) sintering at 400°C. The red dashed box highlights the formation of a continuous phase post‐sintering (scale bar: 100 nm). (I,J) *σ* (panel J) and *S* (panel K) of BST, BST‐ST, and BST‐B‐ST pellets. Reproduced with permission [46]. Copyright 2025, AAAS.

Fredrick Kim et al. developed a binding strategy for Bi_2_Te_3_‐based particles using Sb_2_Te_3_ ChaM ions as an inorganic binder [59]. Figure 4A illustrates this binding mechanism. Rheological analysis confirmed that all prepared TE inks behave as yield‐stress fluids (Figure 4B). The critical role of the ChaM agent is demonstrated by its impact on viscoelasticity: increasing the ChaM content continuously lowered the loss tangent (tan *δ*), indicating a transition toward more solid‐like behavior (Figure 4C). In contrast, the ChaM‐free ink exhibited liquid‐like properties and poor stability. This enhanced elasticity was quantified by a higher recoverable elastic strain in ChaM‐containing inks, confirming the superior structural integrity required for reliable 3D printing.

The strategic use of binding agents plays a critical role in optimizing printed Tes [84]. For instance, Te can function as an intrinsic binder within a BiSbTe matrix, as demonstrated by Ali Newaz Mohammad Tanvir et al*.* (Figure 4E) [83]. Rheological analysis, specifically frequency sweep measurements, confirmed that the Te binder modifies ink properties by enhancing particle interactions, which is evidenced by significant changes in the storage and loss modulus (Figure 4F), which allowed for precise control over ink rheology suitable for blade coating. Consequently, this optimization simultaneously improves the *S* and *σ* while reducing *κ*, leading to an enhanced *PF* across different Te concentrations (Figure 4G).

In a complementary approach, Shengduo Xu et al. developed a reactive binding system for extrusion printing of *p*‐type (Bi, Sb)_2_Te_3_ [46]. Their optimal ink combined Bi_0.5_Sb_1_._5_Te_3_ powder with Bi nanoparticles and Sb_2_Te_4_ ChaM. During sintering, these binders transform into a compositionally matched compound that acts as an in situ solder, chemically bonding the original particles (Figure 4H). This process creates robust intergranular connections and dislocation networks. As shown in Figure 4I,J, the resulting pellets (BST‐B‐ST) exhibit substantially superior TE properties compared to binder‐free inks or those with ChaM alone. The enhanced performance is attributed to this effective interfacial bonding, which significantly improves charge transport and structural integrity.

**FIGURE 5:**
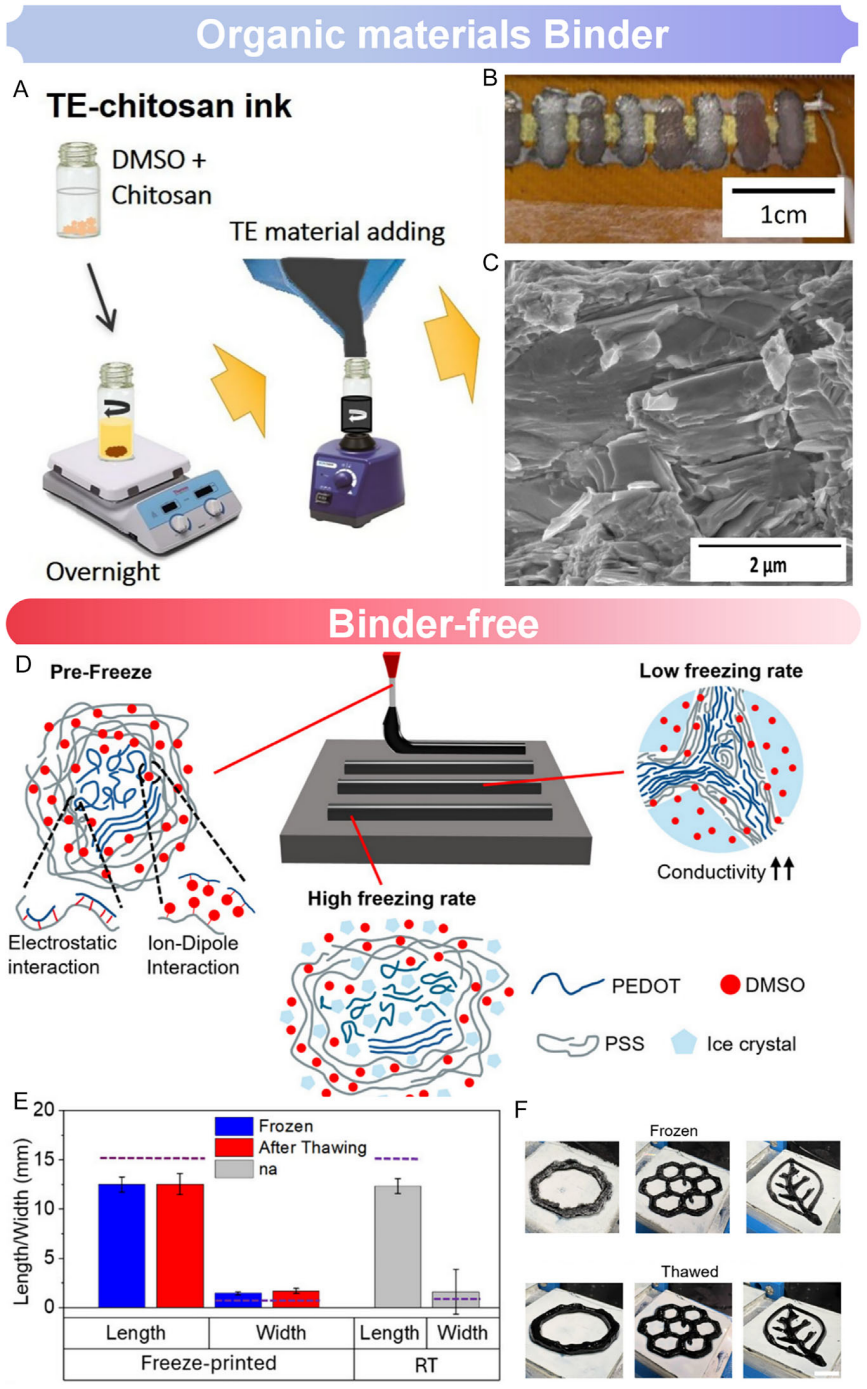
Organic binders and binder‐free printed structures for TE materials. (A) Scheme of the preparation process for BTS‐chitosan composite inks. (B) Optical image of a two‐leg *n*‐type BTS device fabricated by stencil printing. (C) Cross‐sectional SEM image for chitosan‐100 mesh BST. Reproduced with permission [85]. Copyright 2021, Elsevier. (D) Schematic illustration of preparing 3D freeze‐printing of binder‐free PEDOT:PSS. (E) Quantification of the length and width of three‐layered freeze‐printed lines before and after thawing; dashed gray lines indicate ideal length and width. (F) 3D freeze‐printed structures in frozen and thawed states [86]. Copyright 2025, John Wiley & Sons.

As discussed at the beginning of Section 3.2, conventional organic binders often reduce the *σ* of TE films due to their insulating nature. However, selecting appropriate organic materials as binders can mitigate this issue. For example, Eunhwa Jang et al. reported the use of chitosan, a natural and eco‐friendly biomaterial, as an effective binder [85]. Chitosan differs from typical insulating binders (e.g., PVA or PVDF) because its amine and hydroxyl groups can interact with conductive fillers (e.g., CNTs or TE particles) via noncovalent bonding, helping to preserve conductive pathways. In addition, the amount of chitosan used is carefully controlled to remain low enough to avoid forming an insulating barrier. As shown in Figure 5B, a two‐leg *n*‐type BTS TEG was successfully fabricated by stencil printing using chitosan‐based ink. The fabrication process incorporates several key strategies: using a small amount of binder, incorporating TE particles with a broad size distribution, applying low‐temperature and short‐time curing, and employing uniaxial mechanical pressure. These measures help reduce grain boundaries and enhance interfacial connectivity, leading to improved *σ* of the TE composite films. Figure 5C shows the cross‐sectional SEM image for chitosan‐100 mesh BST, displaying the dense structure of chitosan‐BST composite. The combination of minimal natural binder, a heterogeneous particle size distribution, and optimized hot‐pressing conditions resulted in *σ* and *ZT* values comparable to those of other printed TE films, without the need for high‐energy curing. This study demonstrates a sustainable and scalable manufacturing route for high‐performance TEGs, while also showing that with careful binder selection and loading control, organic binders can indeed be promising for printed TEs.

For certain material systems, it is feasible to formulate printable TE inks without adding binders, depending on the intrinsic properties and structure of the material. Joshua Weygant et al*.* [86] used 3D freeze‐printing to fabricate binder‐free, high‐conductivity PEDOT:PSS structures (Figure 5D). By employing a temperature‐controlled plate, they directly extruded dimethyl sulfoxide (DMSO)‐doped PEDOT:PSS solutions, which are typically unprintable via conventional methods due to spreading and poor shape retention, into stable 3D structures. Figure 5E quantifies the length and width of three‐layered freeze‐printed lines before and after thawing, and shows the 3D freeze‐printed structures in both frozen and thawed states.

In addition, Shengduo Xu et al. developed Ag_2_Se (silver selenide) inks for extrusion printing that retain their TE properties after sintering [46]. The printed Ag_2_Se bars undergo a structural evolution during sintering at 350°C for 2 h. Without any binder, the high‐temperature superionic phase of Ag_2_Se exhibits liquid‐like behavior, enabling high Ag^+^ ion mobility and facilitating mass transport across grain boundaries. After cooling to the orthorhombic phase, the Ag–Se atomic arrangement forms a microstructure characterized by interconnected grains and small‐angle grain boundaries. This structure leads to high *σ* and *S*, achieving a *ZT* value of 1.3 at room temperature.

## 4 Fabrication Techniques in Printing TEs

The successful transformation of TE inks into functional devices depends critically on selecting a suitable printing technique and post‐processing protocol. Each printing method provides distinct advantages in resolution, scalability, and compatibility with flexible substrates. To provide a clearer comparison of different printing strategies for TE materials and devices, the key trade‐offs among commonly used techniques are summarized in Table 2, highlighting their respective advantages and limitations in terms of resolution, scalability, film quality, and interfacial properties.

**TABLE 2:** Comparison of major printing methods applied in preparing TEs.

Method	Resolution	Volatility	Cost	Materials density	Contact resistance	Throughput
Inkjet printing	Higher than 10 µm	High	High	High	Low to medium	Medium
Screen print	Higher than 100 µm	Low	Low	Medium	Medium to high	High
DIW	Variable (device‐dependent)	Variable	Variable	Low to medium (varies)	Medium to high	Low
Fused deposition modeling (FDM)	Low‐medium (≈100–500 µm)	Low	Low	Low‐medium	High	Low‐medium
Digital Light Processing	High (≈5–50 µm)	Medium	Medium	High (after sintering)	Low	Medium‐High
Aerosol jet print	Variable, range between 10 and 100 µm	Variable	High	High	Low	Low

### 4.1 Inkjet Printing

Inkjet printing presents considerable advantages for fabricating thin‐film devices, including low cost, digital design flexibility, and minimal material waste [87]. As a noncontact, additive technique, it enables the precise deposition of functional micro‐ and nanomaterials, establishing it as a prominent method for creating customized, high‐precision electronics [88]. Consequently, it is highly suitable for rapid prototyping. However, this method requires low‐viscosity inks with carefully controlled rheology to ensure stable droplet formation and jetting. Achieving high micrometer‐scale resolution remains a significant challenge, primarily due to the limited availability of TE inks that combine high electronic performance with optimal printability. Therefore, developing innovative ink formulations that offer excellent processability, stability, and controlled charge and thermal transport is essential for advancing next‐generation printed TEs. In this context, inkjet printing has been successfully utilized to fabricate devices such as flexible TEGs with graphene‐based legs and generators based on metal chalcogenide NWs like Ag_2_Te [37]. These often employ post‐printing treatments, such as hot‐press sintering, to enhance material crystallinity and final TE performance [89].

**FIGURE 6:**
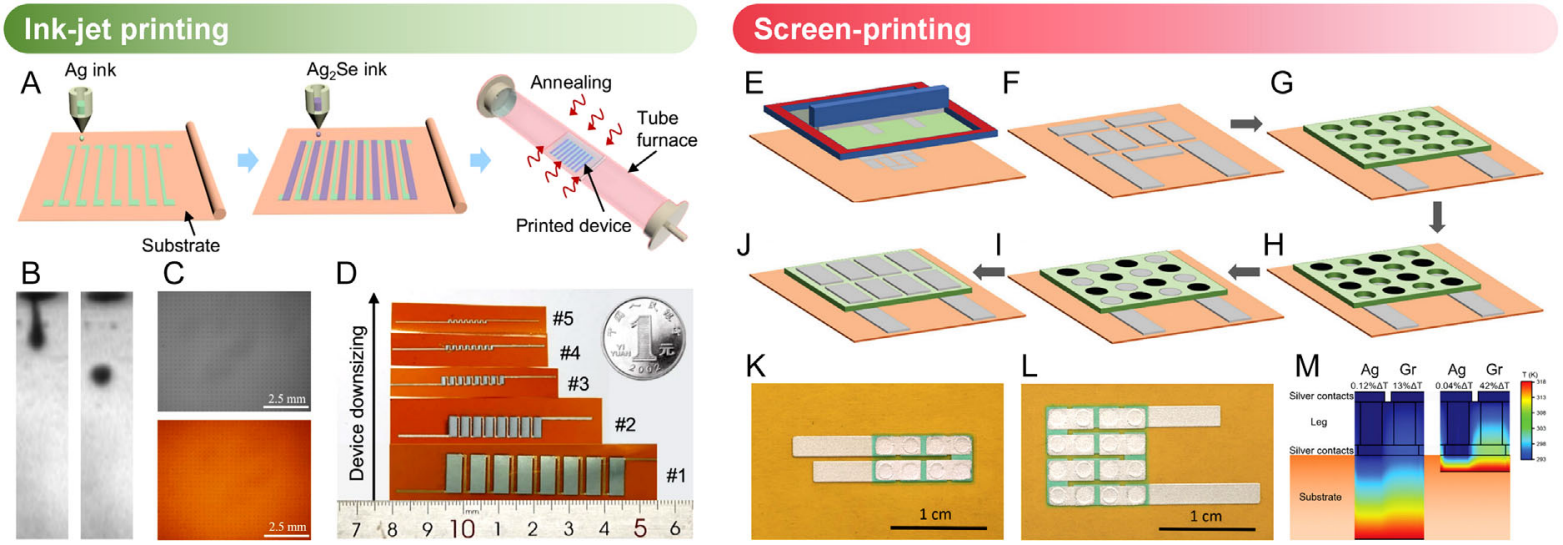
Fabrication processes of printing TE devices by inkjet‐printing: (A) Schematic diagram illustrating the fabrication process for fully inkjet‐printed Ag_2_Se flexible devices. (B,C) Optical images of inkjet‐printed Ag_2_Se droplets on (B) photographic paper for enhanced contrast and (C) polyimide substrates. (D) Digital photographs of fabricated devices showing TE legs with consistent geometric ratios but varying dimensions from micrometers to millimeters. Reproduced with permission [68]. Copyright 2024, Springer Nature. Fabrication processes of printing TE devices by screen‐printing: (E–J) Schematic diagrams and corresponding layers illustrating the screen‐printing fabrication sequence: (E) printing technique overview, (F) bottom silver contact layer, (G) insulator layer, (H) *p*‐type material legs (graphene/PEDOT:PSS), (I) silver legs, and (J) top silver contact layer. (K,L) Photographs of completed screen‐printed TEGs. (M) Cross‐sectional temperature distribution analysis of graphene‐based devices with different substrate thicknesses (25 and 5 µm), showing individual temperature drops (Δ*T*) across silver and graphene legs. Reproduced with permission [90]. Copyright 2024, John Wiley & Sons.

For example, Wan Jiang et al. demonstrated that optimized ink formulations and printing parameters enable the fabrication of large‐area patterned arrays with microscale resolution. Figure 6A illustrates the fabrication process for fully inkjet‐printed Ag_2_Se flexible devices, while Figure 6B shows how droplet morphology depends on cartridge voltage. Figure 6C displays printed Ag_2_Se droplets on different substrates, and Figure 6D presents devices with TE legs spanning micrometer to millimeter dimensions. This approach enables diverse applications, particularly in sustainable power generation through harvesting environmental or body heat [68].

### 4.2 Screen Printing

Screen printing is widely regarded as one of the most mature printing technologies, benefiting from extensive development within the electronics industry. The basic process involves five key components: a screen mask (stencil), squeegee, press bed, printing ink, and substrate. This method is particularly suitable for large‐scale industrial production due to its straightforward and cost‐effective preparation process [91]. As a versatile and high‐throughput technique, screen printing is ideal for depositing thick films from viscous, paste‐like inks. It has been successfully applied to fabricate radial‐structured flexible TEGs on polyimide substrates, producing devices capable of generating substantial output voltages for wearable energy harvesting from body heat.

For instance, Mario Caironi et al. employed screen printing to fabricate fully printed, flexible, and scalable organic monolithic TEGs. Figure 6E illustrates the architecture of the screen‐printing fabrication process, while Figure 6F–J detail the deposition of five distinct layers, each defined by a specialized screen mask. Figure 6K, L shows two variants of PEDOT:PSS TEGs with 4 and 8 thermocouples. Figure 6M presents the temperature distribution across graphene‐based devices with different substrate thicknesses (25 and 5 µm), revealing independent temperature gradients along the silver and graphene legs. This approach enables the engineering of TEGs that, through enhanced thermal coupling and advanced TE inks, could potentially power distributed sensors and IoT devices under modest thermal gradients [90].

### 4.3 Extrusion‐Based 3D Printing

DIW, an extrusion‐based 3D printing technology, has emerged as a versatile method for fabricating custom‐shaped TE architectures [92]. This technique provides exceptional geometric control, spatial resolution, and material placement flexibility, making it particularly suitable for producing customized, skin‐integrated devices. However, DIW imposes stringent material requirements, as printable inks must demonstrate balanced rheological properties, colloidal stability, and structural integrity after deposition.

**FIGURE 7:**
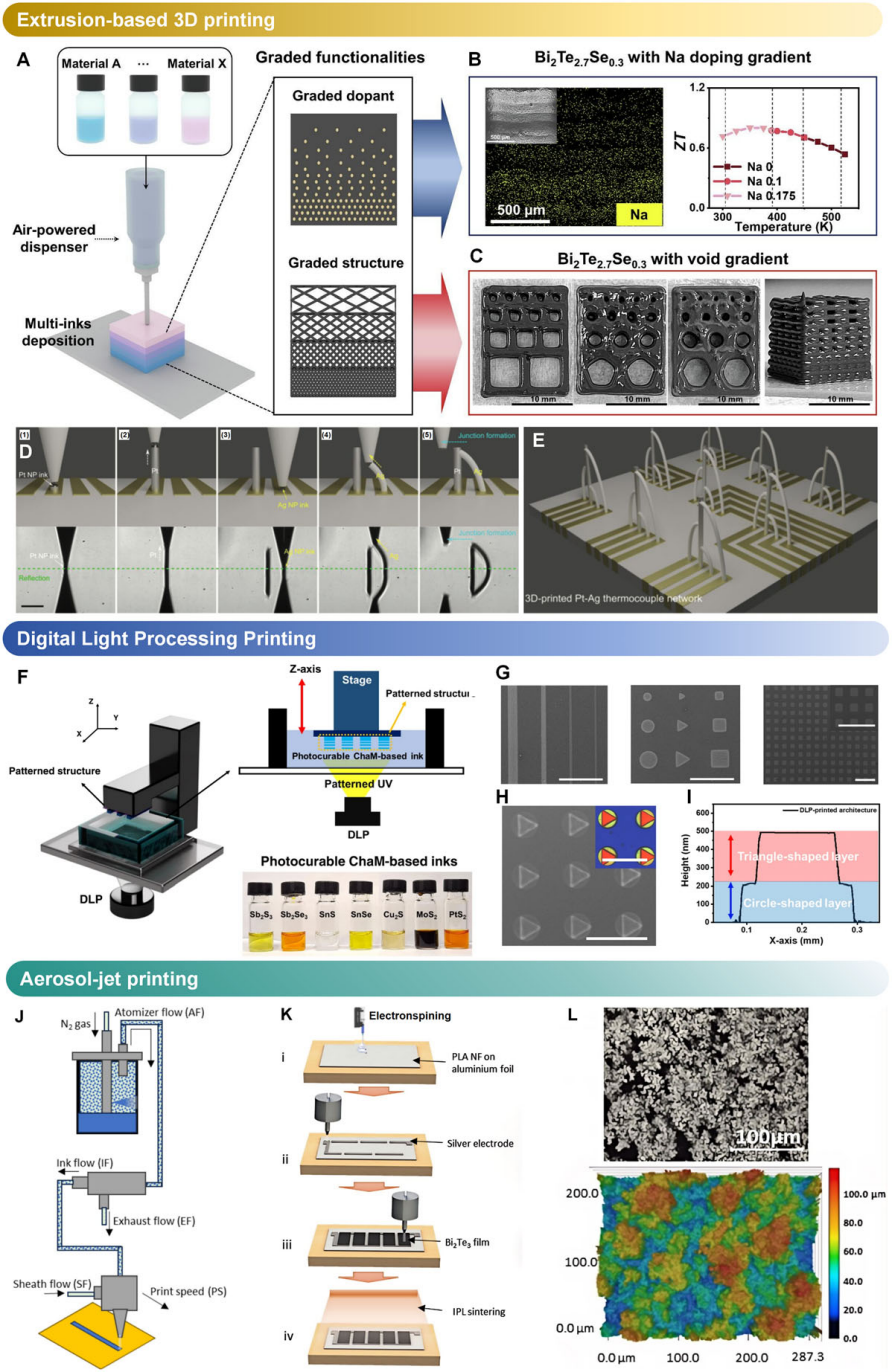
Fabrication processes of printing TE devices by extrusion‐based 3D printing: Macroscopic and microscopic characterization: (A) formulation of viscoelastic Na‐doped Bi_2_Te_2.7_Se_0.3_ lloidal inks, and schematic of DIW. (B) SEM micrographs of 3D‐printed materials and their *ZT*, and (C) complex 3D‐printed architectures by DIW. Reprinted with permission from [93]. Copyright 2024, Elsevier. (D) Schematic illustrations and corresponding optical micrographs of the printing process (scale bar: 20 µm). (E) Design schematic of 3D‐printed thermocouple networks. Reprinted with permission from [94]. Copyright 2023, John Wiley & Sons. Fabrication processes of printing TE devices by Digital light processing (DLP) printing: (F) Schematic of the DLP‐based optical printing process using ChaM inks. (G) SEM images of printed PtS_2_‐based ChaM ink patterns, including line features with widths ranging from 100 to 25 µm, circles, squares, and triangles at the tens‐ to hundreds‐of‐micrometers scale, as well as a large square array composed of hundreds of 100 µm‐wide elements spanning several millimeters. (H) SEM images of a printed 2.5D architecture built from alternating circle‐ and triangle‐shaped layers (inset: 3D scan analysis), and (I) the corresponding height profile of this 2.5D structure. Reprinted with permission from [95]. Copyright 2022, Springer Nature. Fabrication processes of printing TE devices by AJP: (J) Illustration of the AJP mechanism and key operational parameters for controlled material deposition. (K) Schematic of the device fabrication workflow, including electrospun PLA nanofiber substrate preparation, Bi_2_Te_3_ NW printing, and subsequent photonic sintering. (L) Representative microscopy image and corresponding surface topography profile of the resulting printed Bi_2_Te_3_ TE film. Reprinted with permission from [96]. Copyright 2025, Elsevier.

Conventional ink formulations often rely on organic binders to achieve suitable viscoelasticity. Han Gi Chae and Jae Sung Son et al. developed a binder‐free approach for micro‐TEGs. Through precise size control and surface oxidation, they engineered (Bi, Sb)_2_(Te, Se)_3_‐based colloidal inks with inherent viscoelasticity, enabling direct writing of complex 3D architectures. Figure 7A illustrates the DIW setup, where viscoelastic ink is extruded through a nozzle following CAD designs [93]. Figure 7B,C demonstrates printed filaments with varying parameters. Figure 7D,E presents a 3D lattice structure fabricated through layer‐by‐layer deposition [94]. Despite challenges such as volume shrinkage during processing, the method maintains high reproducibility. The resulting micro‐TEGs achieve substantial temperature gradients and a power density of 479.0 μW cm^−2^, demonstrating the potential of DIW for advanced thermal energy conversion applications [90].

FDM printing is another extrusion‐based 3D printing process that builds three‐dimensional objects by extruding a thermoplastic filament through a heated nozzle, melting it, and depositing it layer by layer onto a build platform, where the material solidifies upon cooling. It is one of the most common material extrusion‐based 3D printing techniques, widely used for prototyping and functional parts due to its low cost and material availability. In the field of TEs, Z. Viskadourakis et al. [97] used commercially available PLA‐based nanocomposite filaments, such as PLA‐graphite and PLA‐graphene, to fabricate millimeter‐scale samples, which proved that FDM can be applied for printing TE devices.

### 4.4 Digital Light Processing (DLP) Printing

DLP is an AM technique based on vat photopolymerization. It builds three‐dimensional objects by projecting ultraviolet (UV) light patterns onto a photosensitive resin or ink using a digital micromirror device (DMD). The exposed liquid photopolymer solidifies layer by layer, enabling high‐resolution, mask‐less fabrication with fast printing speeds. DLP is widely used for applications requiring fine features and smooth surface finishes.

Recently, DLP‐based optical printing has been applied to the fabrication of TE materials and devices. In a representative study, Jae Sung Son and colleagues reported a mask‐less DLP printing technology using photocurable ChaM‐based inorganic inks with a photoacid generator. This approach enables the fabrication of organic‐free, patterned 2D metal chalcogenide films and micrometer‐thick 2.5D architectures via layer‐by‐layer AM [95]. Figure 7F–I summarizes the fabrication processes of printed TE devices using DLP‐based optical printing [95]. As shown in Figure 7F, the DLP technique employs ChaM‐based inks to achieve mask‐less, high‐resolution printing. Figure 7G presents SEM images of printed PtS_2_‐based ChaM ink patterns, demonstrating line widths ranging from 100 down to 25 µm, various geometric shapes (circles, squares, triangles) at the tens‐ to hundreds‐of‐micrometers scale, and a large square array of hundreds of 100 µm‐wide features spanning several millimeters. Beyond two‐dimensional patterns, Figure 7H shows SEM images of a 2.5D architecture composed of stacked circle‐ and triangle‐shaped layers (inset: 3D scan analysis), with the corresponding height profile given in Figure 7I. Finally, the complete fabrication route for a microscale TEG via DLP‐based optical printing can be realized based on the above technique. Collectively, these studies highlight the capability of DLP printing to produce complex, organic‐free inorganic chalcogenide structures for TE applications. This design was then realized through finite element simulations and the 3D printing of ternary silver chalcogenide‐based TE materials (AgBiSe_2_ as n‐type and AgSbTe_2_ as *p*‐type), which enables the creation of complex heat‐dissipating architectures via DLP [98].

### 4.5 AJP

AJP is a maskless, digitally controlled AM technique that fabricates micro‐/nanoscale patterns by atomizing functional inks into aerosol droplets, transporting them via a carrier gas, and focusing the aerosol stream with a sheath gas through a nozzle onto a target substrate for direct deposition [99, 100]. As AJP offers broad compatibility with diverse ink viscosities, improved resistance to nozzle clogging, and precise conformal deposition on various substrates, it represents a promising manufacturing strategy for a wide range of TE device fabrication.

Guo Liang Goh et al. demonstrate the scalable fabrication of flexible TE devices via AJP of Bi_2_Te_3_‐based NW inks onto a PLA nanofiber substrate, followed by optimized intense pulsed light (IPL) sintering. The schematic in Figure 7J–L illustrates a representative AJP strategy for fabricating flexible TE devices. As shown in Figure 7J, the AJP process operates by atomizing Bi_2_Te_3_ NW ink into fine aerosol droplets, which are transported by a carrier gas and subsequently focused by a sheath gas through a nozzle for high‐resolution, maskless deposition. This gas‐assisted focusing mechanism enables precise patterning while reducing nozzle clogging issues commonly encountered in conventional inkjet printing. Figure 7K further demonstrates the integrated fabrication workflow, where Bi_2_Te_3_ NWs are conformally deposited onto a flexible PLA nanofiber/aluminum substrate. The microscopy image and surface height map in Figure 7L reveal a relatively uniform and continuous TE thin film morphology, confirming the capability of AJP for controlled layer‐by‐layer deposition. This work highlights AJP as a promising strategy for flexible waste‐heat energy harvesting and next‐generation wearable IoT electronics [96].

## 5 Wearable Applications of Printed TE Devices

### 5.1 Power Generation

**FIGURE 8:**
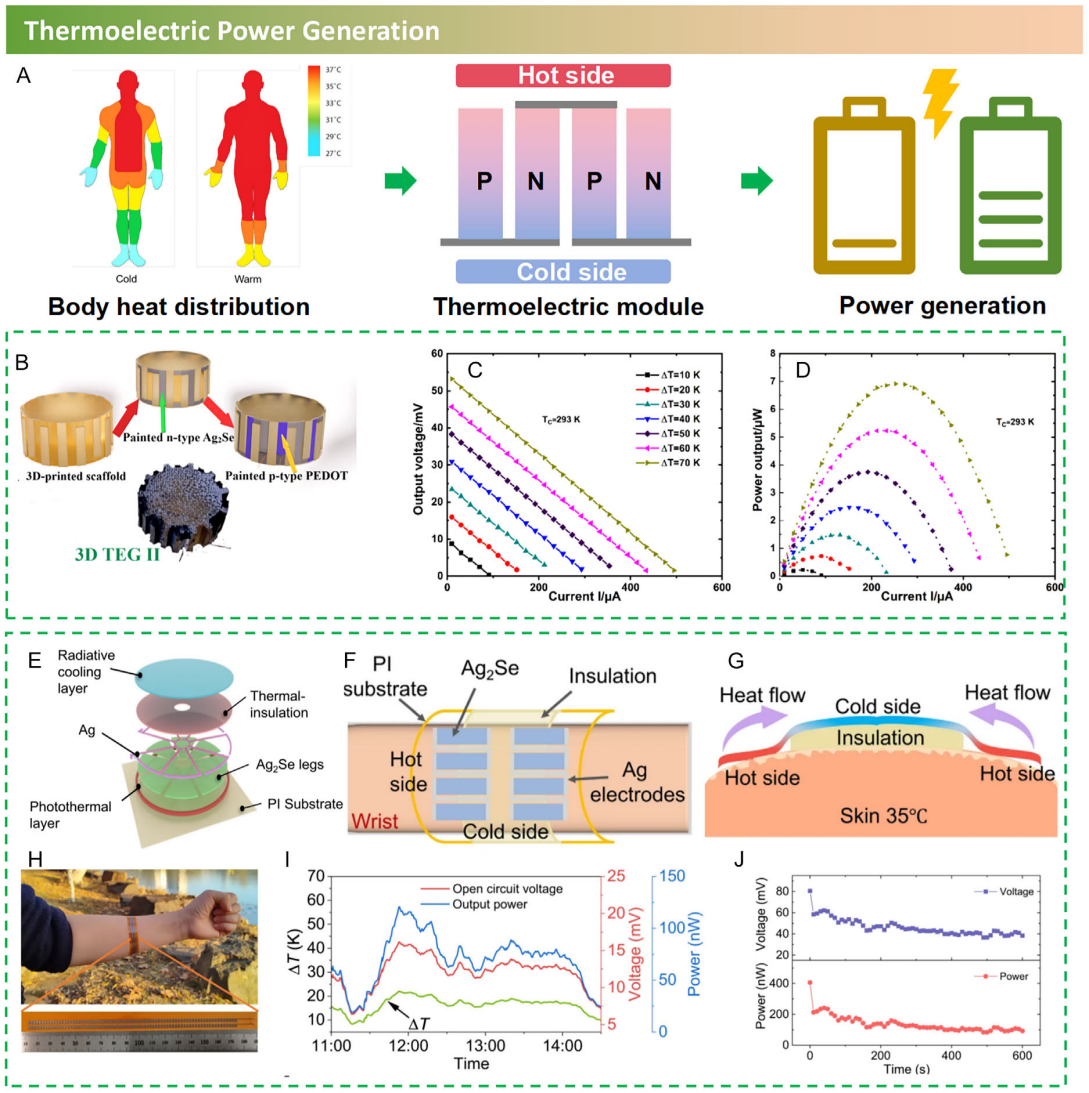
Applications of printed TE devices in power generation. (A) Schematic of a flexible TE device harvesting energy from the human body heat. Reproduced under terms of the CC‐BY 4.0. license [102]. Copyright 2024, Mata‐Romero M. E. et al*.*, published by MDPI. (B) Fabrication process and structural designs of three 3D‐printed TE generators (3D‐TEGs). (C,D) Output voltage and power as a function of current for 3D‐TEG II under temperature differences (Δ*T*) from 10 to 70 K. Reproduced with permission [103]. Copyright 2021, American Chemical Society. (E) Exploded view of a solar thermal/TE/radiative cooling (STR) hybrid device. (F,G) Top and side views of a TEG designed for body heat harvesting. (H) Photograph demonstrating electricity generation using the temperature difference between a wrist and the ambient environment. (I) Power generation performance of the STR hybrid device during daytime operation. (J) Electricity generation performance using body heat. Reproduced with permission [68]. Copyright 2024, Springer Nature.

Printed TE materials and devices show significant potential for advancing wearable electronics, with key applications in energy harvesting, personalized thermal management, and sensing. For energy harvesting, the human body serves as a continuous and renewable heat source [20, 101]. Flexible TEGs can convert the natural temperature difference between skin and the environment directly into usable electricity through the Seebeck effect (Figure 8A) [102]. This capability enables the development of self‐powered systems for health monitoring and other wearable sensors. Such devices, typically fabricated from conformable organic composites, can be seamlessly integrated into textiles or applied directly to skin, offering a viable pathway toward self‐sustaining personal electronics [104, 105].

Md Mofasser Mallick et al. developed a printable n‐type TE ink based on Ag_2_Se that achieves a remarkable *ZT* of approximately 1 at room temperature [103]. Using this optimized ink formulation, they fabricated three TEGs with different architectures through 3D printing technology. As illustrated in Figure 8B, these devices were created by applying the *n*‐type Ag_2_Se ink alongside commercial *p*‐type PEDOT onto 3D‐printed scaffolds, producing TEGs with varying numbers of legs and configurations. Figure 8C,D characterizes the electrical performance of 3D‐TEG II designed in this work, showing the variation of output voltage and power with device current across temperature differences (Δ*T*) ranging from 10 to 70 K.

Compared to 3D‐printed structures, the direct fabrication of two‐dimensional (2D) TE devices on flexible substrates represents a more straightforward application of printing techniques. Wan Jiang et al. demonstrated this approach by developing fully inkjet‐printed, flexible Ag_2_Se TE devices for sustainable power generation from environmental or body heat [68]. This work highlights the potential of such devices to be integrated into emerging electronic systems as sustainable micropower sources. Benefiting from high‐resolution printing, the Ag_2_Se‐based devices achieved a record‐high normalized power density and superior mechanical flexibility. The device architecture is detailed in Figure 8E, which presents an exploded view of a solar‐thermal/TE/radiative cooling hybrid generator. Figure 8F,G shows a device with 150 Ag_2_Se legs designed for body heat harvesting, along with its working mechanism, while Figure 8H demonstrates its wearability on a user's arm. Outdoor testing established a maximum temperature difference (Δ*T*) of 22 K during daytime, resulting in an open‐circuit voltage (*V*_oc_) of 16.1 mV and a maximum output power (*P*_max_) of 120.3 nW (Figure 8I). The output power reached an initial peak of 400 nW before stabilizing at 100 nW (Figure 8J). Thus, the recent progress establishes inkjet printing as a viable method for directly integrating high‐performance TEGs into printed microelectronics [106].

### 5.2 Sensing

In sensing applications, flexible TE devices utilize the Seebeck effect to create self‐powered sensors for advanced bio‐applications such as electronic skin (e‐skin) [107, 108, 109]. These printed, conformal thermocouples enable precise temperature mapping on complex curved surfaces like human skin without requiring external power sources [110]. This capability is essential for e‐skin systems designed to replicate thermal sensation in prosthetics and human–machine interfaces. Additionally, continuous temperature monitoring provides crucial physiological data for personalized healthcare, establishing TE sensing as a fundamental technology for next‐generation wearable diagnostics and interactive robotic systems [111, 112].

**FIGURE 9:**
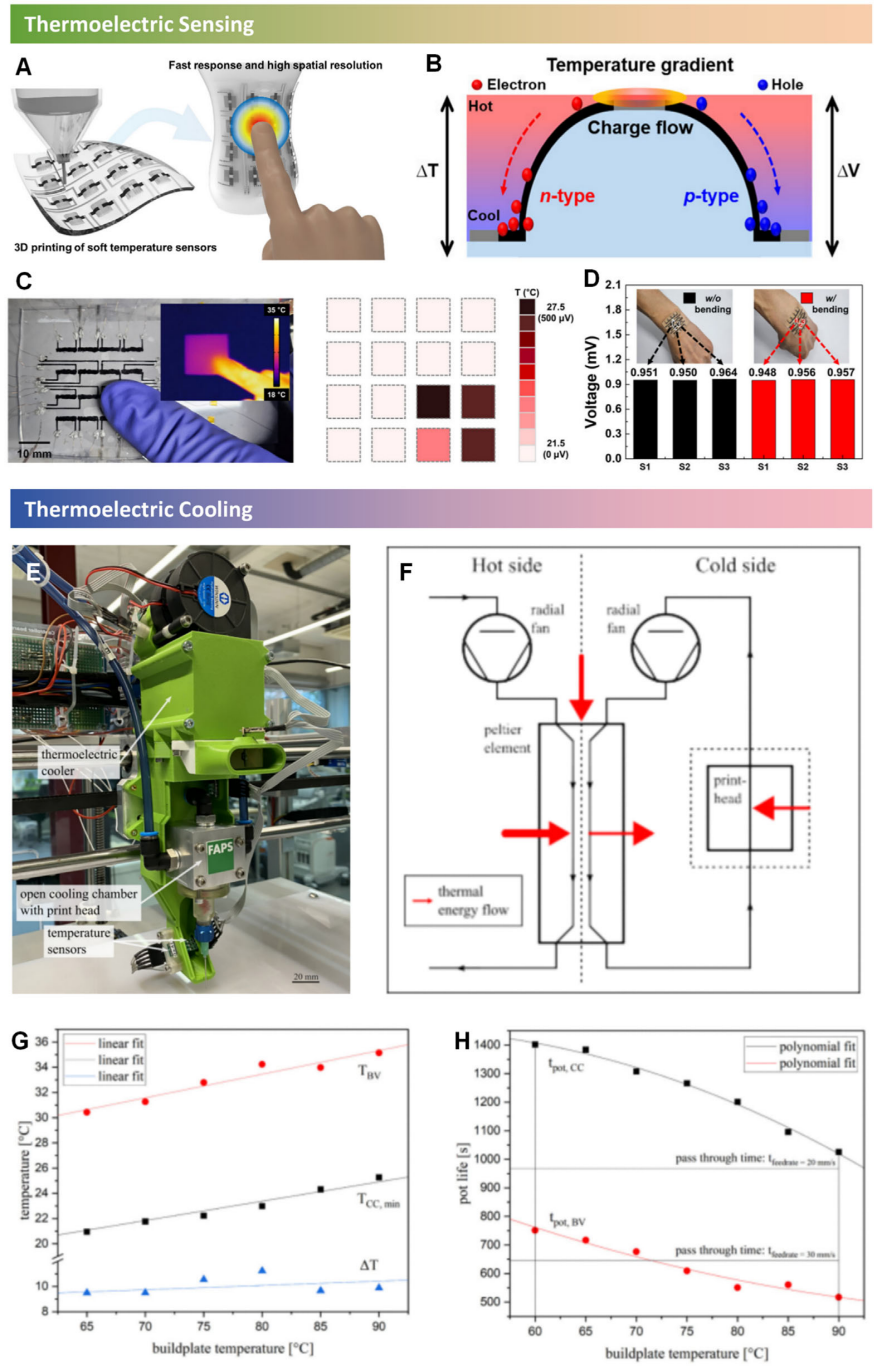
Applications of printed TE devices in sensing and cooling. (A) Schematic and structural illustration of a 3D‐printed soft temperature sensor array. The sensing unit comprises a PDMS substrate, thermal insulation layer, CNT‐based p‐type/n‐type TE legs, undoped CNT electrodes, and PDMS encapsulation. (B) Working mechanism based on the Seebeck effect for temperature detection. (C) Temperature mapping performance on flat surfaces under localized heating. (D) Hot‐spot detection on the human wrist and mechanical stability testing under different bending angles. Reproduced with permission [113]. Copyright 2024, American Chemical Society. (E) Digital photo of the TE printhead cooler. (F) Schematic: The cooling unit uses open and closed air circuits, a Peltier element, and radial fans to transfer heat from the chamber to the exhaust. (G) Instrument performance: Chamber and build volume temperatures rise with build plate temperature, but the chamber stays much cooler. (H) Instrument performance: Higher temperatures reduce calculated pot life; the cooler extends pot life across the tested range. Reproduced under terms of the CC‐BY 4.0. license [114].

Seungjun Chung et al. reported 3D‐printed soft temperature sensors based on TE effects for rapid mapping of localized temperature distributions. These sensors achieve high sensitivity to minor temperature variations, with a minimum resolution of 0.1 K within milliseconds. Figure 9A illustrates the sensor design, while Figure 9B details the sensing unit architecture comprising a PDMS substrate, thermal insulator, CNT‐based *p*‐type and *n*‐type TE legs, undoped CNT electrodes, and PDMS encapsulation. The manufacturing process through full 3D printing is shown in Figure 9C, with the operating mechanism displayed in Figure 9D. The completed 4 × 4 sensor array appears in Figure 9E,F. Experimental validation shows the array's capability for localized temperature detection on both flat (Figure 9G) and curved surfaces (Figure 9H). Furthermore, Figure 9I demonstrates hot‐spot detection on a wrist, while Figure 9j confirms mechanical stability through time‐resolved output voltages during deformation at various angles. This example confirms that printed TE devices enable temperature sensors that accurately map thermal distributions and maintain reliable performance under mechanical stress, benefiting the advanced wearable applications.

### 5.3 Thermal Management

In thermal management, wearable TE coolers utilize the Peltier effect to provide solid‐state, on‐demand temperature control for personal comfort. Unlike conventional cooling systems, these devices contain no moving parts or liquid refrigerants, enabling silent operation and precise temperature regulation [28, 115]. They can be fabricated into thin, flexible patches that conform to skin or integrate into clothing. Furthermore, this technology enables dual‐mode operation, providing either cooling or heating from the same device, making it particularly valuable for advanced wearable systems requiring dynamic thermal management [116]. However, developing effective wearable TE coolers using printed devices remains challenging [117, 118]. A significant obstacle is the required integration of efficient heat sinks, which are often bulky and rigid, conflicting with the desired flexibility and comfort for wearable applications in personal thermal management.

To illustrate recent progress in this field, we highlight the work by M. Ibáñez and coworkers. Their study achieved record‐high room‐temperature *ZT* values of 1.42 for *p*‐type (Bi, Sb)_2_Te_3_ and 1.3 for *n*‐type Ag_2_Se through 3D‐printed structures and optimized sintering, subsequently demonstrating a leading cooling temperature gradient of 50°C in air. The cooling performance and stability were systematically evaluated using custom measurement setups. The fabricated TE cooler (TEC) achieved a maximum temperature difference (Δ*T*_max_) of 50°C under no heating load. As the applied heating load increased, the cooling temperature (*T*_c_) rose gradually. The device reached a maximum cooling flux density of 0.87 W·cm^−2^ at zero temperature difference, reflecting a characteristic trade‐off between cooling capacity and temperature gradient. Furthermore, the TEC exhibited excellent cyclic stability, confirming its potential for practical applications [46].

Figure 9E demonstrates that a TE printhead cooler was integrated into the 3D printer to actively regulate the temperature inside the cooling chamber [114]. As displayed in Figure 9F, the system combines a Peltier element with radial fans and both open and closed air circuits to efficiently remove heat from the chamber. Performance evaluation in Figure 9G,H shows that while chamber and build volume temperatures rise with increasing build plate temperature, the cooling chamber remains substantially cooler. This active cooling extends the calculated pot life of the material across the tested build plate temperature range.

## 6 Challenges in Developing Printed TEs for Wearable Self‐Powered Medical Instruments

Printed TE materials and devices are inherently compatible with wearable electronics owing to their light weight, mechanical flexibility, and low‐cost fabrication. Printed TE devices can uniquely address this gap by converting body heat or a small temperature gradient into a stable, low‐voltage electrical output, which can be directly delivered to target tissues without external batteries [119, 120]. While extensive research has focused on their use as wearable TE sensors and energy harvesters (e.g., body‐heat‐powered generators), this review deliberately shifts the perspective toward a less explored yet highly promising direction: biotherapeutic applications, specifically employing printed TE devices as electrical stimulation platforms.

### 6.1 Perspectives of Wearable TEs in Tissue Engineering

Two types of TE devices have been developed for wearable medical applications: flexible solid‐film generators and soft, conformable TE hydrogels [121, 122]. This externally applied electric field (EF) emulates the function of endogenous bioelectricity, promoting key healing processes including angiogenesis, cell migration, and proliferation [123, 124].

As a representative example of solid‐based TE treatment devices, Chou et al. reported a self‐powered, flexible, and biocompatible TEG constructed from Bi_2_Te_3_‐based compounds [125]. This device exhibits exceptionally high power generation efficiency, producing a voltage of 10 mV under a temperature gradient of 10 K, surpassing all previously reported flexible TE systems. The device was fabricated by alternately depositing *n*‐type Bi_2_Te_3_ and *p*‐type Bi_0.5_Sb_1.5_Te_3_ layers, each approximately 150 µm thick, onto a flexible polyimide substrate via magnetron sputtering (indicated by black arrows), resulting in a circular device architecture. Furthermore, in vivo animal studies validated the therapeutic potential of the device, indicating that the applied electrical stimulation significantly accelerates wound healing.

A representative example of a hydrogel‐based TE treatment device was recently reported by Lu Wang, Lei Wei, et al. Their system employs a thermogalvanic cell (TGC) dressing, which harnesses the temperature gradient between the wound and the dressing to generate an endogenous‐like EF [126]. This EF actively promotes wound healing by guiding and accelerating the migration of epithelial cells. The TGC dressing produces an exogenous EF, where *T*_c_ and *T*_h_ denote the temperatures at the cold and hot sides, respectively, and *φ* represents the electric potential. The use of solid and hydrogel TE materials provides a promising method for creating devices that treat wounds with electrical stimulation [127]. A particularly important step forward is that these materials can now be used to make functional inks for printing. This means we can use printing methods, like ink‐jet or 3D printing, to create these devices. Printing allows us to produce flexible and custom‐shaped devices that fit perfectly against the uneven surface of the skin. This good contact is crucial for efficiently using the body's own heat to generate electricity [128]. For this hydrogel‐based application, printing techniques capable of producing irregular shapes are preferred. For example, extrusion‐based 3D printing allows the deposition of high‐viscosity inks containing either solid TE particles or hydrogel precursors [129], enabling the construction of thick, multilayered, and conformable structures.

### 6.2 Flexibility of Wearable Printed TEs

Mechanical robustness is essential for printed TEDs, especially in flexible sensing and biomedical settings. These devices must continue functioning reliably under cyclic bending, compressive forces, and strain during routine usage. At present, printed TEDs lack sufficient flexibility and mechanical endurance for real‐world applications. Addressing this requires strategies such as microstructural tailoring, composite material design, and substrate engineering to boost mechanical resilience and ensure long‐term operational reliability. Although limited reports display the flexibility performance of representative printed TE devices and their potential application scenarios. TE devices obtained by various techniques, Table 3 shows the flexibility performance of recently reported printed TE devices and their potential application scenarios.

**TABLE 3:** Flexibility performance of representative printed TE devices and their potential application scenarios.

Major material system	Print technique	Flexibility assessment	Key device performance	Estimated applications field
CNTs [113]	DIW	*V*/*V*_0_ of <±3% during 9 cycles	A minimum sensing resolution of 0.1 K	Temperature sensors
Bi_2_Te_3_ [130]	3D printing (laser powder bed fusion)	A change in the internal resistance of less than 60% after bending 500 times to a 7.5 mm radius around the *y*‐axis, and 20% around the *x*‐axis	*P* *F* *s* above 1500 µW m^−1^ K^−2^	Self‐powered sensing and energy harvesting
CNTs [49]	Inkjet printing	No significant changes in the device resistance and output voltage under mechanical deformation, even after 3000 bending cycles at a bending radius of 18 mm	TEG with 600 PN pairs generates unparalleled milliwatt‐scale power at Δ*T* = 25 K	Self‐powered wearable electronics
Ag_2_Se [68]	Inkjet printing	The resistance of the device Increases by less than 10% after 3000 cycles	High normalized power (2 µW cm^−2^ K^−2^)	Self‐powered wearable electronics

### 6.3 Biocompatibility Risks of Wearable Printed TEs

To guarantee the safe use of TE materials in medical settings, their biological interactions must be thoroughly characterized. Biocompatibility is a key requirement for any wearable device. Among promising TE candidates, chalcogenide compounds such as SnSe, Ag_2_Se, Cu_2_Se, Bi_2_Se_3_, SnTe, Bi_2_Te_3_, and Sb_2_Te_3_ have drawn considerable attention. Generally speaking, tellurium‐based alloys tend to be more hazardous than selenium‐based alloys. In 2024, Y.Z. Pei and coworkers [131] conducted a systematic research on the evaluation of chalcogenide compounds applied in TEs by using implants, and found that tellurides releasing Te ions can cause severe cytotoxicity. In contrast, most selenides exhibit better biocompatibility within certain concentrations. This observation agrees with our previous biosafety evaluation of Ag_2_Se, which can be released from hydrogel wound dressings [132]. Thus, inherent rigidity and potential long‐term toxicity risks of these inorganic TE materials still raise concerns for direct skin contact or implantable wearable devices.

In contrast, polymer‐based TE materials (*e.g.*, conducting polymers like PEDOT:PSS composited with inorganic nanostructures) may offer superior biocompatibility and mechanical flexibility, owing to their organic matrix that minimizes the leaching of toxic ions and better mimics the mechanical compliance of biological tissues. However, a critical limitation remains that the TE performance of these polymer‐based systems lags significantly behind that of inorganic counterparts, which restricts their practical application in wearable electronics.

## 7 Conclusion

TE materials, capable of direct thermal‐to‐electrical energy conversion, present a compelling pathway for sustainable energy technologies. Despite substantial advances in the material *ZT*, a critical challenge remains in developing scalable and flexible manufacturing processes for fabricating devices with varied geometries. AM, particularly printing, presents a promising pathway by enabling precise geometric control and scalable fabrication of high‐performance TE devices, whose properties are fundamentally governed by the formulated TE inks. This review has systematically examined progress in TE inks through three interconnected domains: Material Preparation, fabrication techniques, and applications, mainly in wearable electronics, highlighting advanced devices for energy harvesting, thermal management, and sensing. In addition, printed TEs have great promise for powering wearables, IoT sensors, and waste heat recovery systems. Their low‐cost, flexible, and scalable nature enables seamless integration into curved or portable electronics, paving the way for self‐powered devices in smart buildings, healthcare monitoring, disease treatment, and industrial energy harvesting.

## Author Contributions

**Juxiang Shao**: conceptualization, methodology, writing – original draft, review & editing, writing – original draft, investigation, data curation. **Lin Li**: writing – original draft, investigation, data curation. **Ming Yang**: visualization, literature survey, data curation. **Jiali Huang**: visualization, literature survey, data curation. **Na Zhang**: visualization, literature survey, data curation. **Qiang Sun**: visualization, investigation, data curation. **Hongchun Luo**: visualization, investigation, data curation. **Hao Wu**: supervision, writing – review & editing.

## Funding

This work was supported by Open Research Fund of Computational Physics Key Laboratory of Sichuan Province, Yibin University (No.YBUJSWL‐KX‐2025‐01); Natural Science Foundation of China (Grant No. 32501206); Sichuan Science and Technology Program (Grant No. 2026NSFSC0366).

## Conflicts of Interest

The authors declare no conflicts of interest.

## Data Availability

Data sharing not applicable to this article as no datasets were generated or analyzed during the current study.
